# Infants in Control: Rapid Anticipation of Action Outcomes in a Gaze-Contingent Paradigm

**DOI:** 10.1371/journal.pone.0030884

**Published:** 2012-02-17

**Authors:** Quan Wang, Jantina Bolhuis, Constantin A. Rothkopf, Thorsten Kolling, Monika Knopf, Jochen Triesch

**Affiliations:** 1 Frankfurt Institute for Advanced Studies, Frankfurt, Germany; 2 Department of Psychology, Goethe-University Frankfurt, Frankfurt, Germany; Indiana University, United States of America

## Abstract

Infants' poor motor abilities limit their interaction with their environment and render studying infant cognition notoriously difficult. Exceptions are eye movements, which reach high accuracy early, but generally do not allow manipulation of the physical environment. In this study, real-time eye tracking is used to put 6- and 8-month-old infants in direct control of their visual surroundings to study the fundamental problem of discovery of agency, i.e. the ability to infer that certain sensory events are caused by one's own actions. We demonstrate that infants quickly learn to perform eye movements to trigger the appearance of new stimuli and that they anticipate the consequences of their actions in as few as 3 trials. Our findings show that infants can rapidly discover new ways of controlling their environment. We suggest that gaze-contingent paradigms offer effective new ways for studying many aspects of infant learning and cognition in an interactive fashion and provide new opportunities for behavioral training and treatment in infants.

## Introduction

As an infant tries to make sense of the vast array of signals from its sense organs and wins control over its body and physical environment, one of its most fundamental problems is to learn which sensory events are the consequence of its own motor actions and which ones are not, in other words, to discover agency. It has been difficult shedding light on this ability in infants because of their limited motor repertoires [1], [2]. Fortunately, however, infants reach accurate control over their eyes comparatively early [3], [4], suggesting that eye movements could be used as a window into their ability to discover novel action outcomes. Using a newly-developed gaze-contingent (GC) paradigm employing automated eye-tracking, we here show that 6 and 8-month-old infants readily look at targets to trigger certain sensory events and that they rapidly anticipate the outcomes of their actions. In contrast to previous paradigms for studying infant cognition based on looking behavior [5]–[12], our paradigm gives infants direct control over the physical environment, allowing them to change what is “out there” with their eye movements. Such gaze-contingent paradigms based on eye-tracking have been explored with adult subjects before [13], but only recently it has become possible to apply eye tracking to infants [12], [14]. The ability of infants to quickly discover new ways of controlling their environment that we demonstrate here, paves the way for truly interactive new paradigms for studying infant learning and cognition and may provide a basis for novel training and medical intervention strategies.

## Results

**Figure 1:**
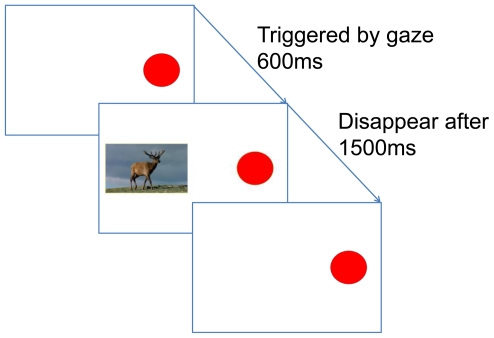
Design and timing of Experiment 1.

In Experiment 1, infants learned to look at a red disc on a screen in order to trigger the appearance of animal pictures (Video S1). Subjects were twenty-four 6-month-olds (17 female, 7 male) and six 8-month-olds (3 female, 3 male). The computer screen initially displayed only the red disc (Fig. 1). By looking at this “button” the infant triggered a brief “bing” sound as well as the appearance of an animal picture, which was displayed adjacent to the red disc. Sound and picture occurred with a delay of 600 ms after the infant had looked at the disc. The animal picture stayed on the screen for 1.5 s before it disappeared and the infant could trigger the button again after one second to drive the appearance of a new animal picture.

**Figure 2:**
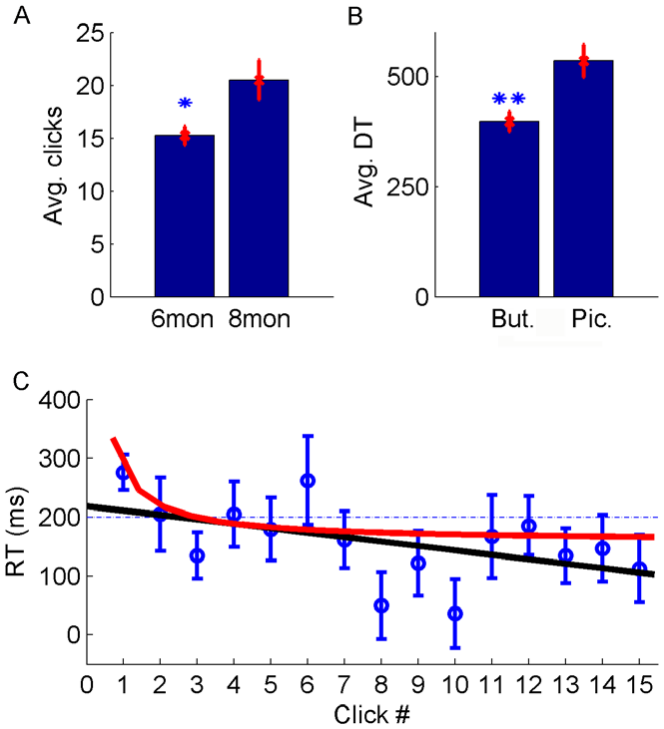
Results of Experiment 1. (A) Average click counts of 6-month-olds and 8-month-olds. (B) Average fixation durations on image area and button area. (C) Average reaction time as a function of click number (error bars indicate s.e.m.). Only the first 15 clicks were plotted, since only a minority of infants performed 16 or more clicks. Infants' average reaction time after their first click is 277 ms, but becomes much faster by the third click. A linear curve (y = −7.53*x+218.77, 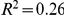) and an inverse curve (y = 123.5/x+158.35, 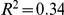) were fitted to infants' average reaction times.

**Figure 3:**
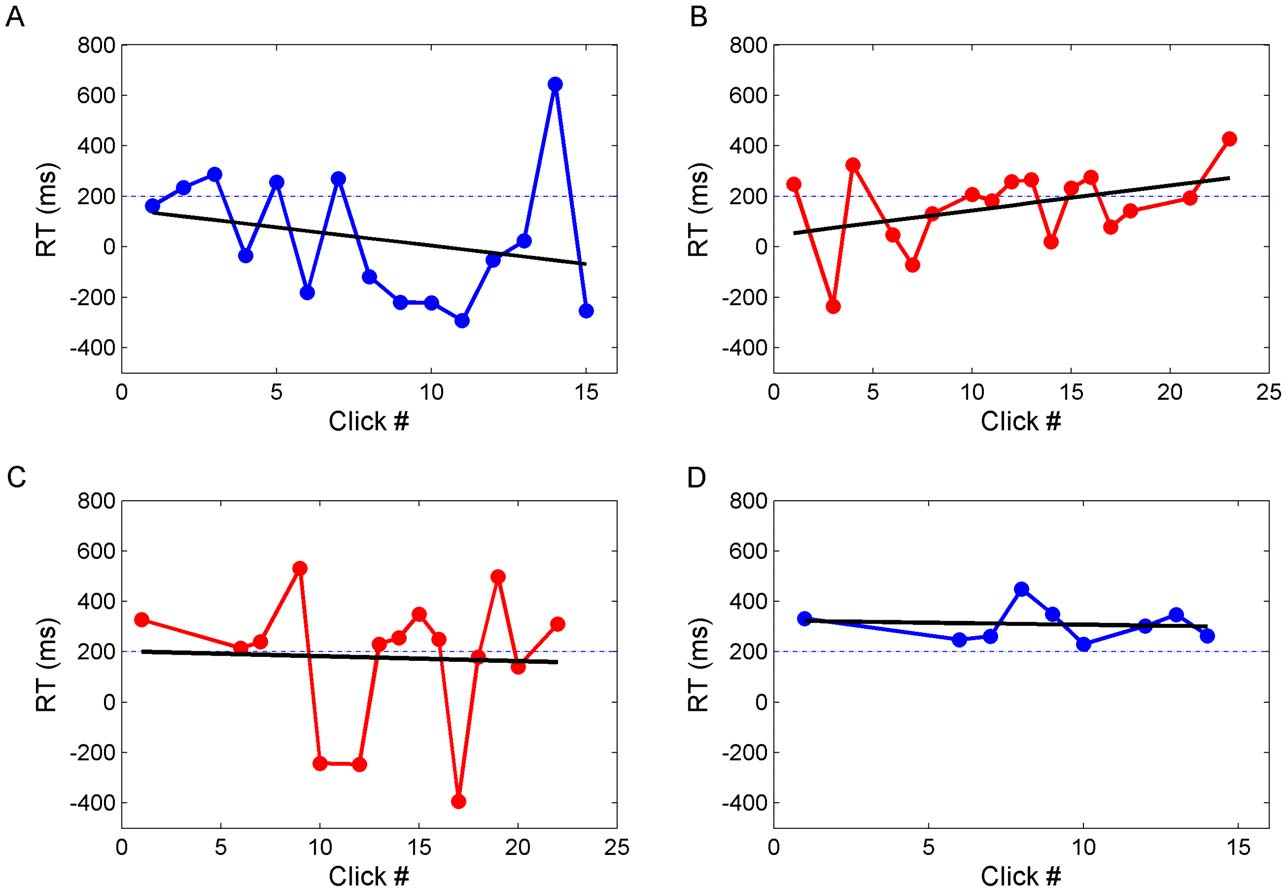
Individual reaction times in Experiment 1. Each subplot represents one individual. Six-month-olds are plotted in blue, eight-month-old in red. Dashed horizontal lines are the anticipation threshold of 200 ms. There was no systematic difference between age or gender groups. Four representative individuals were selected and ordered based on their average reaction times. (A) Very frequent anticipations. (B) Frequent ancitipations. (C) Few anticipations. (D) No anticipations.

**Figure 4:**
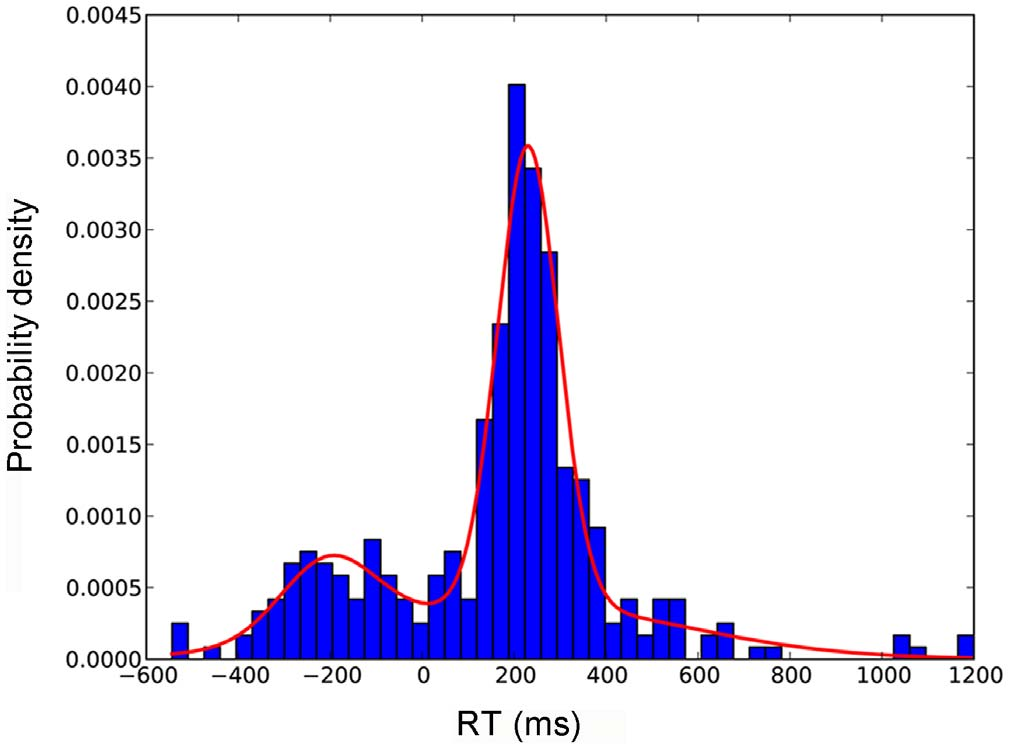
Three-component Gaussian mixture model fitted to reaction times. We found a strong component corresponding to reactive gaze shifts (mean 230 ms, variance 66 ms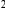, explaining 53% of the data), a weaker component for anticipations (mean −204 ms, variance 103 ms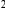, explaining 14% of the data), and a third component of much larger variance covering outliers (mean 219 ms, variance 348 ms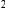, explaining 33% of the data).

Within a minute infants frequently “clicked” the button, with 8-month-olds doing so significantly more often than 6-month-olds (Fig. 2A, one-way-ANOVA, p = 0.017). An analysis of fixation durations on the button vs. the animal pictures revealed that infants exhibited longer fixations on the animal pictures although these were only present for brief 1.5 s intervals, while the button was present for the entire duration (Fig. 2B, two-tailed t-test, p = 0.0004). This suggests that infants did not merely look at the button because it was highly salient per se, but because they wanted to trigger its function of producing a new animal picture. To further investigate this issue, we analyzed the reaction times of infants to see if they were predicting the appearance of the animal picture. To this end the start time of the gaze shift bringing their eyes to the area of the new animal picture was compared to the onset time of the picture. The standard criterion of labeling gaze shifts as anticipatory if they start within 200 ms of the picture onset was applied [8]. Infants of both age groups had 48% of anticipations according to this criterion. When comparing the reaction times of the first click to the subsequent two clicks, we found that infants across both age groups showed a significant decrease in reaction time (one-way ANOVA, Dunnet post hoc p = 0.037). Linear and inverse linear trend lines were fitted revealing decreased average reaction times with increasing number of clicks (Fig. 2C). This suggests that infants rapidly discovered the contingency between looking at the button and the appearance of a new picture. There were large individual differences in reaction times, however. Figure 3 shows reaction times of four representative infants showing very many to no anticipations. Overall, the distribution of infants' reaction times had a bimodal structure with a strong peak for reactive saccades and a smaller one for anticipatory saccades and was well fit by a Gaussian mixture model (Fig. 4). In sum, our data show that most infants produced gaze shifts anticipating the consequence of their new form of agency within a few trials.

**Figure 5:**
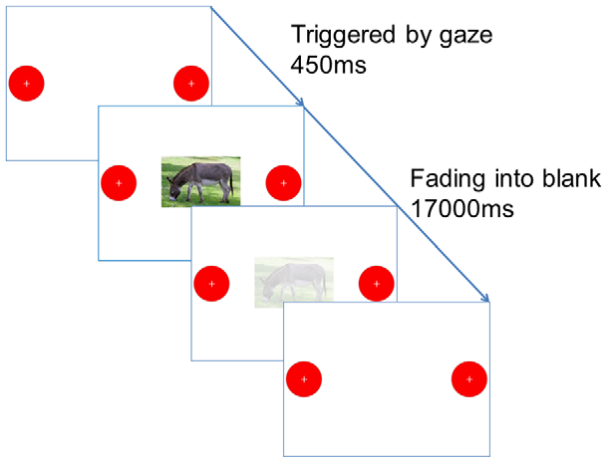
Design and timing of Experiment 2.

In Experiment 1, infants might have merely looked at the red disc because it was the only stimulus left on the screen once an animal picture had disappeared. To address this issue, we designed a second experiment with two modifications. First, two identical red buttons were displayed on either side of the screen. A small cross was added to the red buttons to direct infants gaze towards their center. Importantly, only one of the buttons had the function of triggering the sound and the appearance of a new picture (Fig. 5). The side of the functional button (left or right) was counterbalanced across subjects. Second, the animal picture did not disappear after 1.5 s but slowly faded out over an interval of 17 s. Thus, after the first click, the screen generally contained three objects: the functioning and non-functioning buttons and the fading animal picture. The latency between the triggering of the functioning button and the appearance of a new picture was 450 ms, somewhat shorter than for Experiment 1.

**Figure 6:**
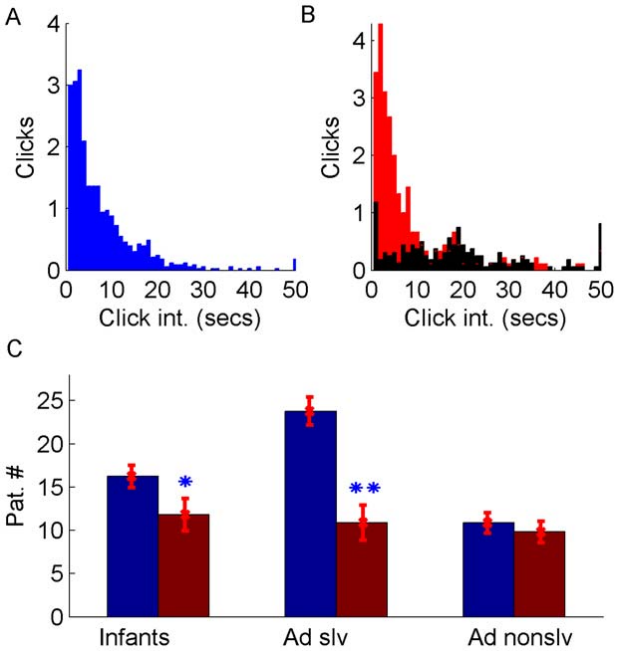
Results of Experiment 2. (A) Click interval distribution of infants. (B) Click interval distribution of adult solvers (red) and non-solvers (black). (C) Pattern count of infants, adult solvers and adult non-solvers (blue: functioning-button pattern, red: non-functioning button pattern).

Subjects were seventeen 6-month-olds (7 female, 10 male) and sixteen 8-month-olds (7 female, 9 male). We also recruited a group of twenty-five adult participants (20 female, 5 male, average age 26 years, range 19 to 49 years). The adult participants (without instructions) were tested with the same experimental procedure as the infants. The experiment ended when thirty pictures had been seen or 5 min had passed. Subsequently, adult subjects filled in a questionnaire testing their understanding of the function of the two buttons. Somewhat surprisingly, the questionnaires revealed that only 9 adults (36%) fully understood the function of the buttons. We divided the adults into two corresponding groups, solvers and non-solvers. For both groups we evaluated the distribution of click intervals, i.e., the periods between subsequent clicks on the functioning button (Fig. 6A, B). Click intervals of adult solvers were significantly different from those of the adult non-solvers (Wilcoxon rank sum test, p = 1.65e-20), with only the adult solvers showing many click intervals shorter than 10 s. Interestingly, the data of the infants (Fig. 6A) closely match the data of the adult solvers who had understood the function of the buttons (p = 0.35), but differs significantly from the data of the adult non-solvers (p = 2.1e-29). To test for specific differences in the usage of the two buttons we further analyzed eye movements by considering the frequency of two gaze patterns: a sequence of saccades leading from the picture area to the (non-)functioning button and back to the picture area was labeled a (non-)functioning-button-pattern. Both the infant group and the adult solvers showed a significant preference for the functioning-button-pattern over the non-functioning-button-pattern (Fig. 6C; paired-sample t-test, infants, p = 0.042; adult solvers, p = 0.006). This was not the case for the adult non-solvers (Fig. 6C; p = 0.885).

## Discussion

Our data suggest that 6 and 8-month-old infants can quickly discover novel forms of agency. They learn to manipulate their environment using their eyes in a gaze-contingent (GC) paradigm by selecting fixation targets that produce certain sensory outcomes and they rapidly anticipate the outcomes of their actions. Previous approaches to studying instrumental conditioning in infants were limited by the comparatively crude and stereo-typed motor skills that they considered including sucking and leg kicking [1], [2]. The central advantage of the GC paradigm is that it taps into a large repertoire of discernible actions (eye movements to various objects or locations, or possibly eye blinks) that infants can perform accurately at a very young age. With our method we could demonstrate rapid anticipation of action outcomes in infants as young as 6 months, while the earliest previous report of such behavior was a recent study showing 10-month-olds anticipating the consequence of a manual button press [15]. Our findings also raise many further questions. For example, how will these results vary as a function of infant age or the delay between looking at the button and the onset of sound and picture? More generally, our method provides a paradigm to effectively investigate central issues of discovery of agency and instrumental learning in infants. In an independent recent study of Deligianni et al. [16], an experimental condition has been realized in which a presented object became animated when an infant fixated this object long enough, giving another example of how gaze contingency can be used in infancy research.

It is interesting to note that in Experiment 2 our group of 6- and 8-month-old infants performed better than the large group of adult non-solvers. We speculate that the adult subjects have learned over many years that just looking at inanimate objects does not produce any effects in the external world. Infants, however, lack this extensive experience and may be more ready to infer a causal connection between their looking and changes in their physical environment.

What may be the physiological basis of the learning processes leading to the discovery of agency? We speculate that infants' ability to rapidly anticipate the consequences of their actions may be related to a recent proposal that the short-latency dopamine signal, which is triggered by unexpected salient sensory events, serves the discovery of novel actions [17]. In our paradigm such a signal might be triggered by the initially unpredicted appearance of the animal pictures evoked by eye-movements acquiring the button. Further experiments are needed to shed light on this issue. Note that such a mechanism for discovering agency may also play a central role in mastering social interactions, where the infant needs to discover that its caregivers and other conspecifics react contingently to its behavior [18].

In general, GC paradigms based on eye tracking technology may have a number of advantages compared to classic non-eye-tracking paradigms for studying infant learning and cognition. First, they extract very rich and detailed behavioral data. Second, they allow studying various aspects of infant cognition in an interactive fashion, giving young infants, who are very restricted by their language and motor abilities, the possibility to communicate with and act on the outside world. Third, by putting infants in control of their environment, GC paradigms are likely more engaging and satisfying for the infant. In fact, infants displayed frequent signs of positive affect in the experiments. The lower attrition rates in infant-controlled over experimenter-controlled habituation paradigms [19] are also suggestive of a greater satisfaction in paradigms where the environment reacts contingently on the infant. Fourth, the use of GC paradigms may allow testing young infants in adaptations of many classic instrumental learning paradigms used in the animal learning literature. Fifth, GC paradigms may be used to train cognitive abilities in infants [20], allowing early intervention in populations at risk. For these reasons, we expect to see many new GC paradigms in infancy research in the future.

## Materials and Methods

Families were recruited from a database of parents who had expressed an interest in participating in research by calling back in response to an information flyer distributed locally. The Ethics Committee of the German Psychological Society only requires ethical approval for interventional studies that involve potential harm to the subject. In this study, no intervention was applied on the subjects as the task only involved looking at a computer screen, as a result no ethical approval was necessary. Informed written consent was obtained by all parents. Infants received a small toy for their participation. All infant participants were healthy and their birth week, weight, and Apgar-score reached standard values [21]. Infants were tested within 10 days of their 6-month or 8-month birthday.

Experiments were performed in a darkened and sound-attenuated room, with the eye tracker screen as the only source of lighting. Infants sat in an infant-seat (Weber Babyschale, http://www.weber-products.de) placed on their parent's lap. An EyeLink 1000 remote eye-tracking system was used (SR Research, http://www.sr-research.com). The eye tracker camera was attached underneath a 17 inch computer screen, and recorded the reflection of an infrared light source on the cornea relative to the pupil at a frequency of 500 Hz. The experimenter controlled the stimulus presentation from a display computer in an adjacent room while monitoring the infants behavior through a video camera. The eye-tracker allowed for moderate head movements without accuracy reduction in a volume of 22 cm×18 cm×20 cm (horizontal×vertical×depth). Blink or occlusion recovery was faster than 3 ms.

During the calibration process, attractive balls (shrinking from approx. 2.5 to a point) with sound were presented in a three-point calibration sequence for infants, and a five-point-calibration was used with adults. The calibration procedure was repeated if necessary. Calibration procedures, stimulus presentation and data output were accomplished using Experiment Builder software and allowed an optimal accuracy of 0.5 degrees. The x, y coordinates (in pixel) of the three point calibration positions on a screen resolution of 1024×768 were: (511.5, 65.2), (961.6, 701.8) and (61.4, 701.8), for five point calibration they were (395.5, 287.5), (395.5, 526.1), (359.5, 48.9), (675.9, 287.5) and (43.1, 287.5). A human observer pressed a key when the participant was judged to be fixating the stimulus at each location, and an exclusion criterion was a 1.5 degree average error during validation of the calibration, which corresponded to a 1.5 cm area on the screen with a viewing distance of about 60 cm.

Animal pictures were taken from Animal Diversity Web (http://animaldiversity.ummz.umich.edu). In Experiment 1, the size of the pictures was 13.8 horizontally; the red disc was 6.1, and the distance between the border of the picture and the border of the disc was 10.1. In Experiment 2, picture width was 12.4 horizontally, each disc's radius was 5.4, and distances between edge of picture and edge of discs were 3.9 on both sides. Interest areas for eye tracking analysis were defined to exactly match position and size of the red discs and images.

Exclusion conditions were if a participant did not finish 1 min in Experiment 1 or 3 min in Experiment 2 because of fuzziness, excessive movement, sleeping, bad calibration, or software failure. Data analysis was performed with EyeLink DataViewer software and Matlab. In Experiment 1, dropout rate was 14% (5 infants). Three subjects were excluded because of bad eye tracker calibration, one because of fuzziness, and one because of a software problem. In Experiment 2, dropout rate was 35% (18 infants). Fourteen infants were excluded because they did not finish 3 min because of fuzziness, excessive movement, or sleeping, three infants because of bad calibration, and one because of a software problem. Adult dropout rate was 11% (3 adults), because of poor eye tracker calibration.

For each data sample, the eye tracking software computed instantaneous velocity and acceleration and compared these to velocity and acceleration thresholds. If either was above threshold, a saccade signal was generated. The Default Cognitive Configuration was applied. Saccade velocity threshold was 30 deg/s and saccade acceleration threshold was 8000 deg/s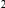. Eye position, pupil size, velocity, etc. were updated every 50 ms during a fixation.

In Experiment 1, anticipatory gaze shifts were identified as follows. Gaze shifts from the button area to the picture area could be composed of an individual saccade or a rapid sequence of two saccades. In both cases, we considerd the start time of the first saccade leaving the button area as the start time of the gaze shift. We only considered situations where the start time of the gaze shift occurred at least 200 ms after the previous image had disappeared to rule out the possibility that the gaze shift was aimed at the previous image. A gaze shift was considered anticipatory if its start occurred within 200 ms of the onset of the new image.

## Supporting Information

Video S1 **Eye movement record of an infant in Experiment 1.**
(WMV)
